# Follicular fluid concentration of soluble Human-G Leukocytic Antigen (sHLA-G) in in vitro fertilization cycles of women with and without peritoneal endometriosis

**DOI:** 10.5935/1518-0557.20240012

**Published:** 2024

**Authors:** Glícia Pinheiro Bezerra, Vanesa K Genro, Carlos Augusto B. Souza, João Sabino Cunha-Filho

**Affiliations:** 1 Programa de Pós-Graduação em Medicina: Ciências Médicas da Universidade Federal do Rio Grande do Sul, Porto Alegre/RS, Brazil; 2 Centro de Reprodução Humana Insemine, Porto Alegre/RS, Brazil; 3 Serviço de Ginecologia e Obstetrícia Hospital de Clínicas de Porto Alegre, Porto Alegre/RS, Brazil

**Keywords:** endometriosis, sHLA-G, follicular fluid, embryonic quality and GES evaluation criteria

## Abstract

**Objective:**

The objective of this research is to investigate the association between the concentrations of soluble human leukocyte G antigen (sHLA-G) in the follicular fluid (FF) in infertile patients with peritoneal endometriosis submitted to in vitro fertilization.

**Methods:**

We performed a cross-sectional study, including ninety-six women undergoing in vitro fertilization (IVF) ageing ≤ 40 years. Infertile patients were classified into two groups: with endometriosis diagnosed by laparoscopy and without endometriosis due to tubal factor. ELISA measured soluble HLA-G in the FF of a pool of punctured (more than 17mm) follicles from women with endometriosis and without endometriosis who were subjected to ovulation induction for IVF. Embryos obtained after fertilization were classified according to the graduated embryo score (GES).

**Results:**

Groups were comparables in terms of age, the number of follicles, AMH, FSH and all included reproductive outcomes. There was no association between sHLA-G concentrations and the average score of the generated embryos (*p*>0.05). Measurement of sHLA-G in the follicle fluid in women with endometriosis and without endometriosis (tubal factor) showed no significant difference (*p*>0.05). We also compared sHLA-G per follicle and per embryo, which were not different between both groups (*p*>0.05).

**Conclusions:**

Patients with peritoneal endometriosis submitted to IVF did not demonstrate an altered sHLA-G in the follicular fluid compared to the follicular fluid sHLA-G concentration in tubal factor patients. Also, this molecule was not linked to any other reproductive outcome.

## INTRODUCTION

Endometriosis is characterised by the presence of endometrial glands and stroma outside the endometrial cavity and the uterine musculature. While its pathogenesis remains unclear, this disease has been extensively studied in recent years and is one of the leading causes of the impairment of female fertility. Moreover, assisted reproduction techniques (ART) are one of the methods capable of reversing infertility in women with endometriosis.

Recently, some investigators described the same reproductive results in patients with endometriosis submitted to IVF compared to non-endometriotic patients, despite the number of related abnormalities in those infertile patients with endometriosis described in the literature (Giudice, 2010; Opøien *et al.* 2012; Barbosa et al., 2014).

Soluble human leukocyte antigen-G (sHLA-G) is a molecule described to be linked to immunological tolerance of the semi-allogeneic fetus at the maternal-fetal interface during pregnancy as well as the activation of NK cells and even embryo quality response (Hunt *et al*., 2000; Noci et al., 2005; Jee et al., 2011; Rizzo et al., 2011; Wunder et al., 2013).

sHLA-G has an important role in the activation of natural killer (NK) cells, Moreover, some authors associated sHLA-G in patients with endometriosis (mainly, the more severe forms) and adenomyosis (Hunt *et al.*, 2000; Wang et al., 2008; Rached et al., 2019). However, the literature is completely absent in terms of the role of sHLA-G in infertile patients with endometriosis and the concentration of this molecule in the follicular fluid during controlled ovarian stimulation for IVF in this group of women.

Endometriosis is not associated with altered embryo quality. However, some recente papers described that granulosa (cumulus) cell function could be altered in these women with peritoneal endometriosis submitted to IVF (De Conto *et al.,* 2017; 2021; Caran et al., 2021).

sHLA-G was also described as a marker for oocyte and embryo quality, and some authors linked this molecule to pregnancy rates (Rebmann *et al.,* 2010). Furthermore, follicular fluid is a crucial environment to analyse and describe the association of sHLA-G and some reproductive outcomes in infertile patients with endometriosis (Wunder *et al.,* 2013).

Considering the link of endometriosis with granulosa cell dysfunction and the possible role of sHLA-G as a marker for oocyte quality, the rationale of this study is to investigate the association between the concentrations of sHLA-G detected in the follicular fluid of infertile patients with peritoneal endometriosis submitted to IVF compared to non-endometriotic patients (tubal factor) as the primary outcome and its effect in some important reproductive outcomes.

## MATERIALS AND METHODS

### Study design

A cross-sectional study was carried out from November 2016 to April 2021 at the Human Reproduction Center.

### Patients

One hundred-nine patients seeking infertility treatment (IVF) for the first time were included.

Eligibility criteria used in the study were age ≤ 40 years, presence of both ovaries, hormone levels of TSH, FSH and PRL within the reference values and indication of in vitro fertilization procedure due to tubal factor or endometriosis, previously diagnosed by video laparoscopy, which was done at least six months before IVF (peritoneal endometriosis confirmed by biopsy and cauterised).

Exclusion criteria were: ovarian hyperstimulation syndrome in the evaluated cycle, autoimmune disease, polycystic ovary syndrome, early luteinisation, endometrioma and presence of blood after follicular fluid centrifugation. Moreover, male partner semen analysis should be normal during semen preparation for IVF.

**Figure 1:**
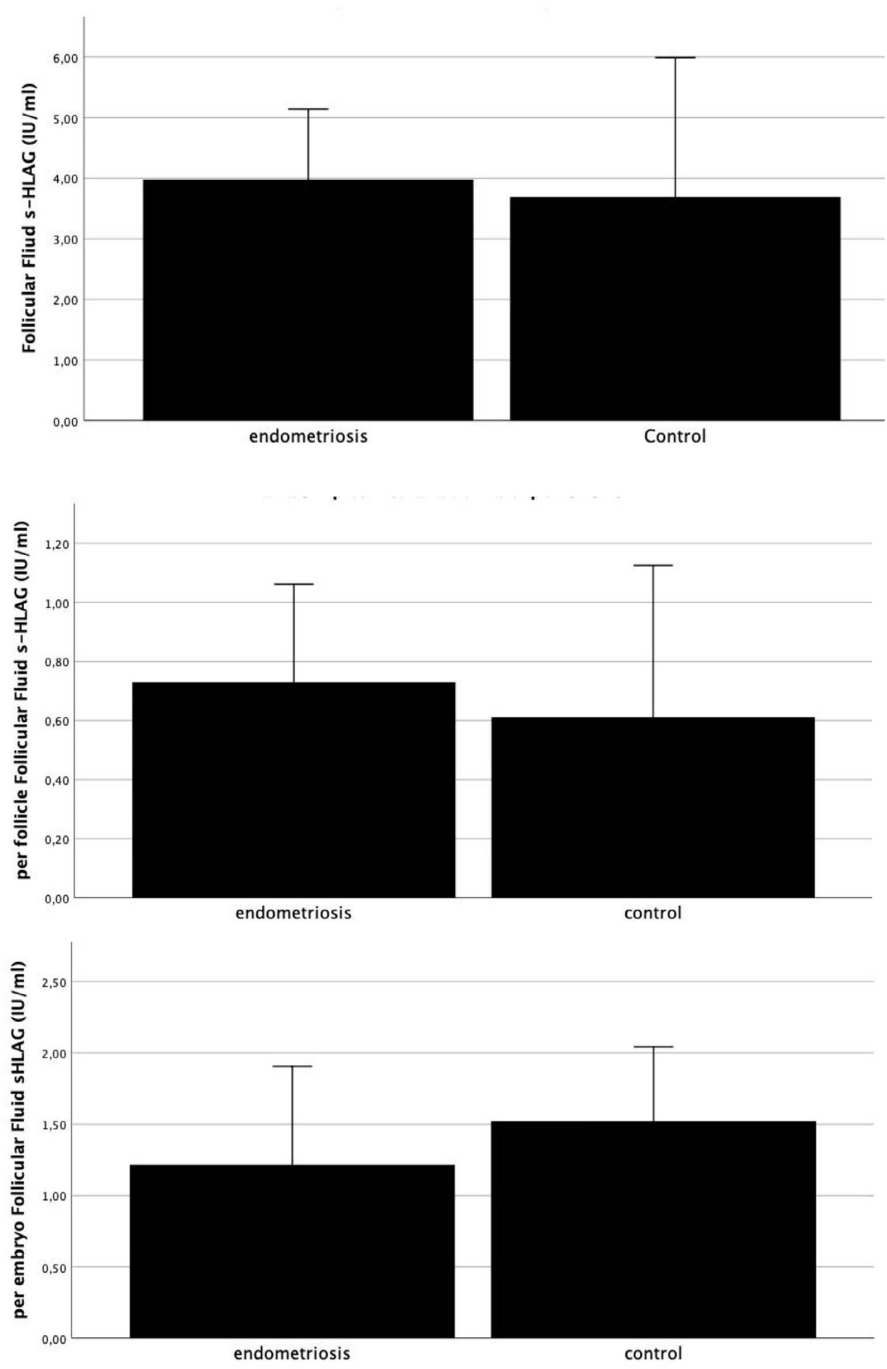
Follicular Fluid concentration of sHLA-G (IU/ml): a) in peritoneal endometriosis vs control group (non-endometriosis), *p*=0.682; b)comparing per follicle sHLA-G concentration between both groups, *p*=0.955 and (c) per embryo sHLA-G concentration (*p*=0.857), Mann- Whitney-U test.

We considered 109 patients with eligibility criteria, 13 patients were excluded; finally, 96 patients were included in this research, as demonstrated in Figure 1. We subdivided into two groups, according to the cause of infertility-that is, with peritoneal endometriosis (n=46) and without endometriosis (n=50) -to assess the association between sHLA-G levels and embryonic quality.

### Diagnosis of Endometriosis

The presence or absence of endometriosis was verified by laparoscopy and biopsy, performed in the last 6 months before in vitro fertilization, and all included patients were diagnosticated with peritoneal endometriosis phenotype according to the European Society of Human Reproduction and Embryology (Kennedy *et al.,* 2005).

### Control Group

The control group was formed by patients with only tubal fator as the infertility etiology. All patients in this group underwent laparoscopy, male analysis, hormonal screening for thyroid and propactin, regular menstrual cycle (21-35 days), age ≤ 40 years and presence of both ovaries.

### Hormonal dosing

Hormonal dosages TSH, FSH and PRL were requested for the patients to be evaluated before beginning the cycle of assisted reproduction and were determined by chemiluminescence immunoassay (Siemens Om-MA Immulite 2000, Munich, Germany). Serum levels of AMH were determined by and enzyme-linked immunosorbent assay (Beckman Coulter, Inc., Brea, CA, USA).

These results were transcribed from the medical records, and only those whose serum measurement was performed on the third day of the cycle were accepted.

### Ovarian stimulation, oocyte recovery and follicular fluid collection

All patients were submitted to ovarian stimulation according to standard protocols. The therapeutic regimen for oocyte stimulation consisted of a GnRH antagonist for pituitary suppression with the administration of recombinant follicle-stimulating hormone (recombinant FSH) for ovarian stimulation. Follicular growth was monitored by transvaginal ultrasound, and when at least three follicles reached a diameter ≥ 17mm, the administration of chorionic gonadotropin (hCG) was determined to induce ovulation. Transvaginal follicular aspiration was performed 36 hours after the administration of hCG under routine intravenous sedation.

At the time of oocyte laboratory recovery, follicular fluid was collected from a pool of punctured follicles (we only included in this study follicles superior to 17 mm with MII oocytes) for each cycle of the respective patient, centrifuged for 5 minutes at 1300 rpm, and, in the absence of blood, stored at -20°C for posterior analysis.

### Assisted Reproduction Procedures

Conventional IVF (in vitro fertilization) was performed 3 hours post puncture.

Embryo score (GES, Graduated Embryo Score) criteria were used for assessing Embryonic Quality and Calculating the Average Embryo Score Generated). The evaluation of embryonic quality was carried out in three stages, from which scores ranging from 0 to 100 points were obtained. An embryo with 100 points is classified according to this criterion with the best morphological quality (Fisch *et al.,* 2001).

The first moment of evaluation occurs 16-18 hours after IVF. At this moment, fertilization is confirmed by visualising the female and male pro-nuclei (PNs) and the presence of 2 polar corpuscles in the perivitelline space. The second moment occurs 25-27 hours after IVF and evaluates the early cleavage (presence of two cells), and the third moment of evaluation occurs 64-67 hours after IVF. In these three moments, the following parameters were evaluated: cell division and the presence of fragmentation with an embryo with maximum quality presenting 6 to 8 blastomeres of uniform size and no fragmentation at this time.

After classifying the scores for each embryo generated from each cycle, the arithmetic mean between these scores was performed. In this study, the quality of the embryo was assessed by the average embryo score (EMEG) whose calculation was performed as follows: EMEG = (sum of scores of N embryos generated)/N (numbers of embryos generated).

### Analysis of the concentration of soluble HLA-G in follicular fluid

The levels of sHLA-G in the follicular fluid were measured by the enzyme-linked immunosorbent assay (ELISA) technique using the ELISA Kit (RD194070100R, Bio Venda, Czech Republic). The kit with the anti-sHLA-G monoclonal antibody can identify and measure the soluble isoforms HLA-G1 quantitatively (by proteolytic cleavage) and HLA-G5.

Following the recommendations of the kit manufacturer, the samples were not diluted. The 16-20 hour incubation period between the FF sample and the anti-HLA-G monoclonal antibodies is an advantage of the kit since the literature describes considerably shorter periods, and a more extended incubation period allows greater test sensitivity (with a detection limit of 0.6 Units/ml). The plates were read at 450 nm, and the concentration of each sample analysed was calculated by the ELISA (Biotek ELX 800) that builds a calibration curve formed between the absorbance of the sample (Y-axis) and the concentration of the calibrators and samples tested (X-axis). The measured HLA-G concentration value is given in units per mL.

### Ethical approval

The Ethics Committee approved this study under the number: 26453514.1.0000.5327. STROBE guidelines for observational studies were utilised for this research (von Elm *et al.,* 2008).

### Statistical analysis

Data analysis was performed using chi-square or Fisher’s exact tests for categorical data. Continuous variables were compared with Student’s t-test for parametric data, and Mann-Whitney-U was used for non-parametric data. Multiple comparisons were made using linear regression to address potential confounders. Furthermore, for correlations, we utilised Spearmen/Pearson tests.

Seventy patients were required to have an 80% chance of detecting, as significant at the 5% level, a decrease in the primary outcome measure from 4.35 in the control group to 3 in the experimental group (Rizzo *et al.,* 2007).

We also analysed the s-HLAG for follicles (dividing sHLA-G/number of follicles) and sHLA-G per embryo (total number of embryo/sHLA-G). These analyses were performed using the statistical program Statistical Package for the Social Science (SPSS) 21.0, and the data analysis was considered statistically significant when *p*<0.05.

## RESULTS

Both groups were comparable in terms of age, AMH and reproductive outcomes (Table 1). Pregnancy rates were also not different between the groups (*p*=0.945) as the embryo score per transfer or the median embryo obtained per patient (Table 1). The association (Spearman test) between the following variables was analysed: sHLA-G and AMH, age, number of collected MII, number of follicles, number of embryos and embryo score. All analyses did not demonstrate statistical significance (*p*>0.05). Moreover, the multivariable analysis reinforced that sHLA-G was not related to any reproductive variable or outcome (age, number of follicles, number of follicles > 17mm, pregnancy rates, AMH, FSH and number of mature oocytes).

**Table 1:** Clinical characteristics and reproductive outcomes of included patients. Variables showed as mean±standard deviation (parametric data^a^) or median and 95%CI (non-parametric data^b^).

	Endometriosis N=46	Control N=50	statistics
Age (Y)	35.3±2.5	34.4±2.7	0.079^a^
FSH (IU/ml)	8.2±2.5	7.3±3.4	0.256^a^
TSH (mIU/l)	1.9 (1.6-2.2)	1.8 (1.7-2.5)	0.177^b^
AMH (ng/ml)	2.8 (2.4-4.9)	2.0 (2.3-6.4)	0.507^b^
Number of MII	6.0 (4.4-8.0)	5.0 (4.0-7.2)	0.688^b^
Total Number of	3.0 (2.2-4.7)	3.0 (2.3-4.6)	0.950^b^
Embryo Embryo transfer	2.0 (1.4-2.0)	2.0 (1.2-1.9)	0.177^b^
Mean embryo score	58±30	52±31	0.356^a^
Mean embryo score per	62±35	54±36	0.228^a^
transferred embryo Pregnancy rate	41%	42%	0.945^c^

a t-student test;

b Mann-Whitney;

c Chi-square test.

Performing the Mann-Whitney-U statistical test, the amount of sHLA-G measured in the group of women with peritoneal endometriosis and women without endometriosis was analysed, and we found no significant difference (*p*=0.682). We also compared s-HLAG per follicle and per embryo between both groups of patients, and all analyses were not significant (*p*=0.682, 0.955 and 0.857, respectively) (Figure 1).

## DISCUSSION

We demonstrated that peritoneal endometriosis was not associated with an altered sHLA-G follicular fluid concentration. Moreover, sHLA-G was not related to embryo quality, pregnancy rates, AMH, age or endometriosis.

Endometriosis is a heterogeneous disease associated with infertility and one of the main causes of IVF. Furthermore, several mechanisms involving granulosa cells were described for this group of patients, involving prolactin secretion, decreased anti-Mullerian hormone and dysfunction in BMP-6 and SMAD4 in cumulus cells linked to peritoneal endometriosis (Cunha-Filho *et al.*, 2001; 2003; De Conto et al., 2017; 2020). Nowadays the effect of endometriosis during an IVF cycle is disputed and has recently been questioned by several authors, but it is essential to understand the role of this disease during the IVF process to enhance pregnancy rates (Opøien *et al.,* 2012; Barbosa et al., 2014; González-Comadran et al., 2017; Caran et al., 2021). Therefore, we decided to better understand the granulosa cell (follicular compartment) of patients with peritoneal endometriosis submitted to IVF, analysing sHLA-G as a marker for oocyte/embryo competence and development (Rizzo *et al.,* 2007; Jee et al., 2011; Wunder *et al.,* 2013). We included only peritoneal endometriosis because this specific phenotype was already described (Santulli *et al.,* 2016) as the most linked to infertility compared to the other phenotypes of endometriosis (endometrioma/deep).

In this study, we also analysed the association of sHLA-G with endometriosis and with several potential markers of oocyte development and reproductive outcomes. The fact that sHLA-G is not related to endometriosis comes following recent papers showing that endometriosis per se is not a detrimental factor for patients submitted to IVF (Barbosa *et al.,* 2014; González-Comadran et al., 2017). Furthermore, sHLA-G seems to be an independent factor in oocyte competence/embryo quality, as demonstrated in a multicenter study (Rebmann *et al.,* 2010).

Also, previous studies that showed an association between HLA system and endometriosis focused on the peritoneal fluid compartment and the immunological role of HLA molecules in patients with endometriosis. Deep endometriosis is more linked to several immunological abnormalities, and HLA molecules may play a role in the activation of NK cells in this group of patients (Bylińska *et al.,* 2018; Rached et al., 2019; Ścieżyńska et al., 2019). We have demonstrated that the immunological profile of peritoneal endometriosis is different from other phenotypes (deep and ovarian endometriosis), which could partially explain our results (D’Hooghe *et al.,* 2001; Glitz et al., 2009; Andreoli et al., 2011; Carmona et al., 2012).

Follicular fluid sHLA-G concentration was associated with oocyte competence and embryo fertilization but not with good-quality embryos (Jee *et al.,* 2011). Besides, when performing oocyte maturation in close contact with cumulus oophorus complex (COCs), these cells produced sHLA-G during the oocyte maturation process, and sHLA-G was not detected in supernatants in the culture of COCs with immature oocytes, suggesting that sHLA-G is part of the oocyte maturation process but is not the limiting factor in that process (Rizzo *et al.,* 2009).

We included strict criteria for embryo quality (Fisch *et al.,* 2001) to improve our statistical power and understand the relationship between sHLA-G and embryo development. As mentioned before and proved by our multifactorial analysis, sHLA-G is an independent factor (not associated with other reproductive parameters) that has been linked to embryo competence and even pregnancy rates after IVF by one study (Rebmann *et al.,* 2010). However, these authors stressed that embryo morphology is better than sHLA-G to select embryos.

However, another group of authors did not find any association between sHLA-G in pregnant or not pregnant women after IVF (Wunder *et al.,* 2013). Furthermore, our study, when correlating the average score of the generated embryos and the concentrations of sHLA-G in the follicular fluid, concluded that the analysis of sHLA-G in FF is a parameter that is not associated with embryonic quality. This result corroborates with some studies in the literature. Future work could potentially utilise artificial intelligence or neural networks related to sHLA-G to improve embryo selection and better classify/choose the best embryo for transfer (Bormann *et al.,* 2020; VerMilyea et al., 2020).

Our study had several limitations. First, we included only peritoneal endometriosis. Next, some reproductive outcomes need more subjects to be analysed, and for some outcomes, our number included patients was limited (pregnancy rate, for example), we calculated our sample size based on follicular fluid sHLA-G. Besides, sHLA-G is an unpractical tool, supplemented by embryo score and recently the advent of artificial intelligence. We included only superficial (peritoneal) endometriosis to perform a homogeneous group and increase our external validation. The main objective of this study was to investigate sHLA-G. For this purpose, we calculated the sample size, and our research included a sufficient number of patients. However, for reproductive outcomes, we need to include more patients, since analysing reproductive outcomes typically requires systematic reviews or international databases (Barbosa *et al.,* 2014; González-Comadran et al., 2017). The usefulness of sHLA-G during daily life in a human reproduction clinic is limited and obsolete, but it still does not invalidate this utility in terms of a research protocol.

In this study, when analysing the sHLA-G, a kit with a detection limit of 0.6 Units/ml was used and therefore offered a greater sensitivity than the ELISA Kits described in the literature. The increase in this limit is directly related to the long incubation time (16-20 hrs) of the antibodies in contact with the FF samples, making it possible to detect sHLA-G in all the analysed samples in our study. Note that the importance of ELISA sHLA-G accuracy was already stressed by others (Dahl & Hviid, 2012). We, therefore, emphasise the need for a more sensitive ELISA test both for studies with measurements in FF and embryo cultures.

## CONCLUSIONS

We conclude that the level in the follicular fluid of sHLA-G in patients with superficial endometriosis was not altered compared to tubal factor patients submitted to IVF. Thus, we accept the null hypothesis that the concentrations of sHLA-G in follicular fluid in women with and without endometriosis do not differ.

## References

[r1] Andreoli CG, Genro VK, Souza CA, Michelon T, Bilibio JP, Scheffel C, Cunha-Filho JS. (2011). T helper (Th)1, Th2, and Th17 interleukin pathways in infertile
patients with minimal/mild endometriosis. Fertil Steril.

[r2] Barbosa MA, Teixeira DM, Navarro PA, Ferriani RA, Nastri CO, Martins WP. (2014). Impact of endometriosis and its staging on assisted reproduction
outcome: systematic review and meta-analysis. Ultrasound Obstet Gynecol.

[r3] Bormann CL, Thirumalaraju P, Kanakasabapathy MK, Kandula H, Souter I, Dimitriadis I, Gupta R, Pooniwala R, Shafiee H. (2020). Consistency and objectivity of automated embryo assessments using
deep neural networks. Fertil Steril.

[r4] Bylińska A, Wilczyńska K, Malejczyk J, Milewski Ł, Wagner M, Jasek M, Niepiekło-Miniewska W, Wiśniewski A, Płoski R, Barcz E, Roszkowski P, Kamiński P, Malinowski A, Wilczyński JR, Radwan P, Radwan M, Kuśnierczyk P, Nowak I. (2018). The impact of HLA-G, LILRB1 and LILRB2 gene polymorphisms on
susceptibility to and severity of endometriosis. Mol Genet Genomics.

[r5] Caran J, Genro VK, Souza CAB, Cunha-Filho JS. (2021). The Graduated Embryo Score of Embryos from Infertile Women with
and without Peritoneal Endometriosis. Rev Bras Ginecol Obstet.

[r6] Carmona F, Chapron C, Martínez-Zamora MÁ, Santulli P, Rabanal A, Martínez-Florensa M, Lozano F, Balasch J. (2012). Ovarian endometrioma but not deep infiltrating endometriosis is
associated with increased serum levels of interleukin-8 and
interleukin-6. J Reprod Immunol.

[r7] Cunha-Filho JS, Gross JL, Vettori D, Dias EC, Passos EP. (2001). Growth hormone and prolactin secretion after metoclopramide
administration (DA2 receptor blockade) in fertile women. Horm Metab Res.

[r8] Cunha-Filho JS, Lemos NA, Freitas FM, Kiefer K, Faller M, Passos EP. (2003). Insulin-like growth factor (IGF)-1 and IGF binding protein-1 and
-3 in the follicular fluid of infertile patients with
endometriosis. Hum Reprod.

[r9] Dahl M, Hviid TV. (2012). Human leucocyte antigen class Ib molecules in pregnancy success
and early pregnancy loss. Hum Reprod Update.

[r10] De Conto E, Matte Ú, Bilibio JP, Genro VK, Souza CA, Leão DP, Cunha-Filho JS. (2017). Endometriosis-associated infertility: GDF-9, AMH, and AMHR2 genes
polymorphisms. J Assist Reprod Genet.

[r11] De Conto E, Matte U, Cunha-Filho JS. (2021). BMP-6 and SMAD4 gene expression is altered in cumulus cells from
women with endometriosis-associated infertility. Acta Obstet Gynecol Scand.

[r12] D’Hooghe TM, Xiao L, Hill JA. (2001). Cytokine profiles in autologous peritoneal fluid and peripheral
blood of women with deep and superficial endometriosis. Arch Gynecol Obstet.

[r13] Fisch JD, Rodriguez H, Ross R, Overby G, Sher G. (2001). The Graduated Embryo Score (GES) predicts blastocyst formation
and pregnancy rate from cleavage-stage embryos. Hum Reprod.

[r14] Giudice LC (2010). Clinical practice. Endometriosis. N Engl J Med.

[r15] Glitz C, Souza CA, Rodini GP, Genro V, Bilibio JP, Senger M, Cunha-Filho JS. (2009). Peritoneal and serum interleukin-18 levels are not increased in
women with minimum or mild endometriosis. Braz J Med Biol Res.

[r16] González-Comadran M, Schwarze JE, Zegers-Hochschild F, Souza MD, Carreras R, Checa MÁ. (2017). The impact of endometriosis on the outcome of Assisted
Reproductive Technology. Reprod Biol Endocrinol.

[r17] Hunt JS, Jadhav L, Chu W, Geraghty DE, Ober C. (2000). Soluble HLA-G circulates in maternal blood during
pregnancy. Am J Obstet Gynecol.

[r18] Jee BC, Suh CS, Kim SH, Moon SY. (2011). Soluble human leukocyte antigen G level in fluid from single
dominant follicle and the association with oocyte competence. Yonsei Med J.

[r19] Kennedy S, Bergqvist A, Chapron C, D’Hooghe T, Dunselman G, Greb R, Hummelshoj L, Prentice A, Saridogan E, ESHRE Special Interest Group for Endometriosis and Endometrium
Guideline Development Group (2005). ESHRE guideline for the diagnosis and treatment of
endometriosis. Hum Reprod.

[r20] Noci I, Fuzzi B, Rizzo R, Melchiorri L, Criscuoli L, Dabizzi S, Biagiotti R, Pellegrini S, Menicucci A, Baricordi OR. (2005). Embryonic soluble HLA-G as a marker of developmental potential in
embryos. Hum Reprod.

[r21] Opøien HK, Fedorcsak P, Omland AK, Abyholm T, Bjercke S, Ertzeid G, Oldereid N, Mellembakken JR, Tanbo T. (2012). In vitro fertilization is a successful treatment in
endometriosis-associated infertility. Fertil Steril.

[r22] Rached MR, Coelho V, Marin MLC, Pincerato K, Fujita A, Kalil JE, Abrão MS. (2019). HLA-G is upregulated in advanced endometriosis. Eur J Obstet Gynecol Reprod Biol.

[r23] Rebmann V, Switala M, Eue I, Grosse-Wilde H. (2010). Soluble HLA-G is an independent factor for the prediction of
pregnancy outcome after ART: a German multi-centre study. Hum Reprod.

[r24] Rizzo R, Dal Canto MB, Stignani M, Fadini R, Fumagalli D, Renzini MM, Borgatti M, Gambari R, Baricordi OR. (2009). Production of sHLA-G molecules by in vitro matured cumulus-oocyte
complex. Int J Mol Med.

[r25] Rizzo R, Fuzzi B, Stignani M, Criscuoli L, Melchiorri L, Dabizzi S, Campioni D, Lanza F, Marzola A, Branconi F, Noci I, Baricordi OR. (2007). Soluble HLA-G molecules in follicular fluid: a tool for oocyte
selection in IVF?. J Reprod Immunol.

[r26] Rizzo R, Vercammen M, van de Velde H, Horn PA, Rebmann V. (2011). The importance of HLA-G expression in embryos, trophoblast cells,
and embryonic stem cells. Cell Mol Life Sci.

[r27] Santulli P, Lamau MC, Marcellin L, Gayet V, Marzouk P, Borghese B, Lafay Pillet MC, Chapron C. (2016). Endometriosis-related infertility: ovarian endometrioma per se is
not associated with presentation for infertility. Hum Reprod.

[r28] Ścieżyńska A, Komorowski M, Soszyńska M, Malejczyk J. NK (2019). Cells as Potential Targets for Immunotherapy in
Endometriosis. J Clin Med.

[r29] VerMilyea M, Hall JMM, Diakiw SM, Johnston A, Nguyen T, Perugini D, Miller A, Picou A, Murphy AP, Perugini M. (2020). Development of an artificial intelligence-based assessment model
for prediction of embryo viability using static images captured by optical
light microscopy during IVF. Hum Reprod.

[r30] von Elm E, Altman DG, Egger M, Pocock SJ, Gøtzsche PC, Vandenbroucke JP, STROBE Initiative (2008). The Strengthening the Reporting of Observational Studies in
Epidemiology (STROBE) statement: guidelines for reporting observational
studies. J Clin Epidemiol.

[r31] Wang F, Wen Z, Li H, Yang Z, Zhao X, Yao X. (2008). Human leukocyte antigen-G is expressed by the eutopic and ectopic
endometrium of adenomyosis. Fertil Steril.

[r32] Wunder DM, Birkhäuser MH, Bersinger NA. (2013). Soluble human leukocyte antigen-G (sHLA-G) in follicular fluid
and embryo culture medium and its impact on pregnancy prediction in IVF-ICSI
treatment. Immunoanal Biol Spéc.

